# Draft Genome Sequence of Staphylococcus succinus Strain GN1, Isolated from a Basement Floor in Milwaukee, WI

**DOI:** 10.1128/MRA.00580-21

**Published:** 2021-07-08

**Authors:** Grant P. Nickolson, Nasim Maghboli Balasjin, Christopher W. Marshall

**Affiliations:** a Department of Biological Sciences, Marquette University, Milwaukee, Wisconsin, USA; University of Rochester School of Medicine and Dentistry

## Abstract

A strain of Staphylococcus succinus was sampled from the floor of the basement of a house and isolated in an undergraduate classroom in Milwaukee, WI. Here, we report the draft genome sequence of this strain.

## ANNOUNCEMENT

A sterile swab was used to sample the basement floor of a 1916 home in Milwaukee, WI, to see if environmental isolates from a home environment had any measurable antibiotic resistance. A sterile cotton swab was prewetted with sterile water, and then the bare concrete floor of the basement was swabbed. The swab was then immediately placed in the freezer. The next day, the swab was streaked onto brain heart infusion (BHI) agar and incubated at 30°C for 24 h in order to isolate colonies for further experiments and DNA extraction. A single colony, named isolate GN1, was selected at random and streaked twice for isolation. A single colony was then grown in BHI liquid medium for physiological tests and DNA extraction. DNA was extracted using the DNeasy blood and tissue kit (Qiagen). The sample was then sent to the Microbial Genome Sequencing Center for whole-genome sequencing. Libraries were prepared using a modified version of the Illumina Nextera kit as previously described (1) and sequenced on an Illumina NextSeq 550 instrument. The adapters were removed from the 151-bp reads prior to analysis using bcl2fastq. A total of 3,832,226 paired-end reads were recovered. Sequence analyses were done using default settings in the KBase.us narrative interface (2), and the publicly available narrative containing all analyses and data can be found at https://kbase.us/n/51640/53/.

**FIG 1:**
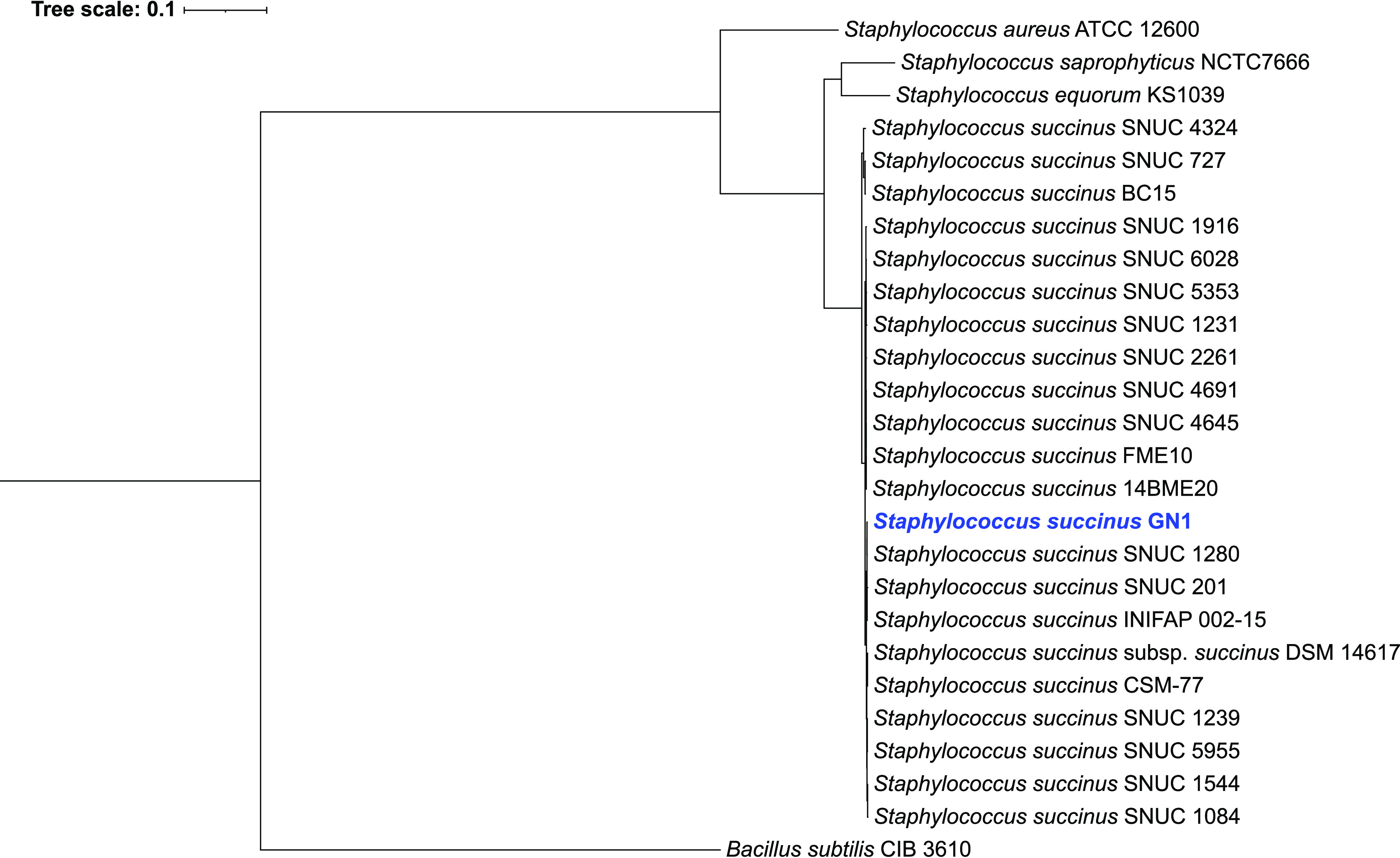
Phylogenetic tree generated using GToTree and IQ-TREE of closely related Staphylococcus succinus assemblies and related Staphylococcus species with Bacillus subtilis as the outgroup. Our isolate, GN1, is indicated in blue. The scale bar indicates substitutions per site.

After the raw reads were uploaded to KBase, SPAdes v3.13.0 was used to assemble the genome sequence (3). The assembly stats compiled by QUAST (4) demonstrate that the assembly contained contigs ranging from 593 bp to 1.4 million bp and had a genome size of 2,838,015 bp and a G+C content of 33%. The assembly consisted of 11 contigs with an *N*_50_ value of 782,976 bp (Table 1). The assembled genome sequence was annotated using the Rapid Annotations using Subsystems Technology (RAST) v0.1.1 toolkit to detect gene locations and functions (5, 6). We also uploaded the assembly to NCBI with the associated BioSample accession number, where gene predictions and annotations were completed using NCBI Prokaryotic Genome Annotation Pipeline (PGAP) (7). The Genome Taxonomy Database (GTDB) v1.1.0 was used for taxonomic identification (8) and FastANI (9) for relatedness. Based on the average nucleotide identity, our isolate was 98.5% identical to Staphylococcus succinus (GenBank accession number GCF_001006765.1). A phylogenetic tree of our isolate, GN1, and the available *S. succinus* genome assemblies (Fig. 1) was created using the GToTree v1.5.46 phylogenomics workflow (10). Single-copy marker genes were identified using HMMER v3.2.1 (11), aligned using Muscle v3.8 (12), and trimmed using trimAl v1.4 (13), and the phylogenetic tree was generated using IQ-TREE v2.0.3 (14). The pipeline was run using GNU Parallel v20201122 (15). Because of the high sequence similarity and placement on the phylogenetic tree, our isolate is likely a strain of *S. succinus* that we will refer to as *S. succinus* strain GN1.

**TABLE 1:** Genome assembly statistics

Statistic	Value
GC%	32.85
Genome size (bp)	2,837,157
No. of contigs	11
*N*_50_ (bp)	1,426,624
No. of predicted coding genes	2,758

Staphylococcus succinus strains are typically aerobic, Gram-positive bacteria that are commonly isolated from ripened cheese (16, 17). Staphylococcus strains are spherical bacteria that aggregate in grapelike clusters (18), consistent with the morphology of our isolate as determined by Gram stain. We tested susceptibilities by disk diffusion to eight different antibiotics (neomycin, kanamycin, tetracycline, ciprofloxacin, bacitracin, streptomycin, chloramphenicol, and ampicillin) and found that isolate GN1 was susceptible to all antibiotics. We also tested Staphylococcus succinus strain GN1 on a limited number of carbon sources and found that it was able to utilize a pyruvate, lactose, or citrate source on either M9 minimal medium (pyruvate, lactose) or Simmons citrate agar (citrate).

### Data availability.

This genome is part of a larger collection of genomes deposited at the National Center for Biotechnology Information (NCBI) under the BioProject accession number PRJNA665534. This is the repository for an undergraduate microbiology laboratory course. Students isolate a single colony, conduct physiological assays, sequence the genome, and conduct bioinformatics analyses. The sequences for isolate GN1 were deposited at the NCBI Sequence Read Archive under BioProject accession number PRJNA665534, BioSample accession number SAMN16337256, and SRA accession number SRR12762898. The assembly accession number is JAHKBG000000000.1. A public narrative was created in KBase that assembled the genome, generated trees, and computed pangenome analyses (2). A static narrative can be found at https://kbase.us/n/51640/53/, which links to the full manipulatable data set. 
